# Depression of corticomotor excitability after muscle fatigue induced by electrical stimulation and voluntary contraction

**DOI:** 10.3389/fnhum.2015.00363

**Published:** 2015-06-19

**Authors:** Shinichi Kotan, Sho Kojima, Shota Miyaguchi, Kazuhiro Sugawara, Hideaki Onishi

**Affiliations:** Institute for Human Movement and Medical Sciences, Niigata University of Health and WelfareNiigata, Japan

**Keywords:** transcranial magnetic stimulation, motor-evoked potential, F-wave, M-wave, fatigue, tetanic electrical stimulation

## Abstract

In this study, we examined the effect of muscle fatigue induced by tetanic electrical stimulation (ES) and submaximal isometric contraction on corticomotor excitability. Experiments were performed in a cross-over design. Motor-evoked potentials (MEPs) were elicited by transcranial magnetic stimulation (TMS). Corticomotor excitability was recorded before and after thumb opposition muscle fatigue tasks, in which 10% of the maximal tension intensity was induced by tetanic ES or voluntary contraction (VC). The participants were 10 healthy individuals who performed each task for 10 min. Surface electrodes placed over the abductor pollicis brevis (APB) muscle recorded MEPs. F- and M-waves were elicited from APB by supramaximal ES of the median nerve. After the tetanic ES- and VC tasks, MEP amplitudes were significantly lower than before the task. However, F- and M-wave amplitudes remained unchanged. These findings suggest that corticospinal excitability is reduced by muscle fatigue as a result of intracortical inhibitory mechanisms. Our results also suggest that corticomotor excitability is reduced by muscle fatigue caused by both VC and tetanic ES.

## Introduction

Muscle fatigue is defined as an exercise-induced decrease in maximum voluntary contraction (MVC) force produced by a muscle or muscle group (Gandevia, 2001; Taylor and Gandevia, 2008). Changes occur at all levels of the motor pathway, including the muscle fiber, muscle fiber membrane, neuromuscular junction, motoneurons and segmental and supraspinal circuits, during and after fatiguing contractions (Taylor and Gandevia, 2001). Moreover, sustained voluntary maximal or submaximal isometric muscle contractions often induce central fatigue and alter corticomotor excitability. Central fatigue has been investigated by many researchers and is evident in an increase in the force evoked by motor nerve stimulation or transcranial magnetic stimulation (TMS) during MVC (Gandevia et al., 1996, 1999; Taylor et al., 1996, 2000a,b,c). Furthermore, several studies have shown that changes in corticomotor excitability occur during and after muscle fatigue induced by maximal or submaximal muscle contractions (Brasil-Neto et al., 1993; McKay et al., 1995; Samii et al., 1996).

Corticomotor excitability can be assessed using TMS and modulated by voluntary muscle contractions or electrical stimulation (ES) delivered to a peripheral nerve. The amplitude of motor-evoked potentials (MEPs) elicited by TMS in relaxed muscles increases during intermittent exercise or immediately after fatiguing exercise compared with that of MEPs evoked before exercise; this transient post-exercise MEP facilitation lasts a few seconds (Samii et al., 1996; Perretti et al., 2004; Benwell et al., 2006). After transient MEP facilitation, MEP amplitude undergoes a prolonged decrease (>30 min) following fatiguing exercise, a phenomenon called post-exercise MEP depression (PED; Brasil-Neto et al., 1993; McKay et al., 1995; Zanette et al., 1995; Liepert et al., 1996; Samii et al., 1996; Gandevia et al., 1999). The duration of PED is related to the intensity and duration of exercise (Samii et al., 1997; Gandevia et al., 1999). It has been suggested that PED is an intracortical phenomenon (Brasil-Neto et al., 1993; Samii et al., 1996). In addition, the mechanism of PED is believed to involve neurotransmitter depletion or increased presynaptic inhibition (Perretti et al., 2004) and decreased excitability of facilitatory cortical circuits (Di Lazzaro et al., 2003). However, the origin of PED after voluntary fatiguing exercise is poorly understood.

ES of a peripheral nerve is commonly used to change corticomotor excitability, and it is established that ES induces transient plastic changes in the primary motor cortex (M1). For example, repetitive ES for 2 h (Ridding et al., 2000) or 30 min (Khaslavskaia and Sinkjaer, 2005) leads to a long-term reorganization of M1, and MEP amplitudes evoked by TMS increase after repetitive ES. This effect persists for >30 min after the cessation of ES (Khaslavskaia and Sinkjaer, 2005). In contrast, Chipchase et al., 2011a) evaluated the effects of six ES paradigms on M1 excitability and showed that only repetitive ES in an on–off mode (cycles of 4 s on and 6 s off) for 30 min results in increasing M1 excitability. Moreover, they showed that M1 excitability decreases slightly when continuous ES is applied for 30 min at an intensity below the motor threshold (MT). Although many researchers have studied the influence of ES on M1 excitability, few reports examine the relationship between M1 excitability and muscle fatigue induced by ES. McKay et al. (1995) studied a change in M1 excitability after muscle fatigue induced by continuous ES and found that MEP amplitude remains unchanged when muscle fatigue is induced by tetanic ES. However, the duration of tetanic ES was approximately 70 s and only five individuals participated in their study (McKay et al., 1995). Muscle afferents are believed to fire under partial muscle ischemia during muscle fatigue, despite a decline in the force generated. It is also established that these muscle afferents alter corticomotor excitability (Taylor et al., 1996; Ridding and Rothwell, 1997; Ziemann et al., 2001; Levy et al., 2002). We hypothesized that corticomotor (M1) excitability decreases after muscle fatigue induced by tetanic ES as PED induced by voluntary fatiguing exercise.

The experiments described in this study were designed to investigate whether corticomotor excitability is altered after muscle fatigue induced by tetanic ES. We accordingly compare changes in MEP amplitude of tetanic ES, prolonged voluntary submaximal isometric contractions that induce muscle fatigue and low intensity prolonged ES (90% of MT) that does not induce muscle fatigue.

## Methods

### Participants

Ten healthy volunteers (seven men, three women; aged 21–22 years) participated in this study. None of the participants had a history of neuromuscular or cardiovascular diseases and all gave their written informed consent to participate. The study conformed to the *Declaration of Helsinki* and *International Code of Medical Ethics* of the World Medical Association and was approved by the ethics committee at the Niigata University of Health and Welfare, Niigata, Japan. Informed consent was obtained from all participants in the study.

### Electromyography

Electromyographic activity was recorded via electromyography (EMG) using surface electrodes placed over the abductor pollicis brevis (APB) muscle of the right hand. The active electrode was positioned over the muscle motor point and the reference electrode was placed over the metacarpophalangeal joint. EMG signals were filtered at 20–500 Hz and sampled at 10 kHz using an analog-to-digital converter (Power Lab 8/30; ADInstruments Inc., Colorado Springs, CO, USA).

### Transcranial Magnetic Stimulation (TMS) and Motor-Evoked Potential (MEP) Recording

TMS was applied using a Magstim 200 magnetic nerve stimulator (Magstim Co., Ltd., Whitland, Carmarthenshire, Dyfed, Wales, UK) with a figure-of-eight-shaped coil (diameter, 95 mm). The coil was placed tangentially to the scalp and held at 45° to the midsagittal line for activating the APB muscle. This site was marked with a skin pencil for future reference. The TMS intensity used was the lowest stimulus intensity that induced MEP with a peak-to-peak amplitude, exceeding 1 mV in the relaxed APB in at least 5 of 10 consecutive trials, and this intensity was kept constant throughout the experiments. MEP amplitude was measured in 12 trials before and after each task and analyzed (Scope for Windows; ADInstruments Inc.), with the exception of the maximum and minimum MEP amplitudes.

### F- and M-wave Recording

Excitability of the spinal anterior horn cells was assessed by F-wave recording from the exercised APB muscle. We simultaneously monitored the functional condition of the neuromuscular structure of APB via supramaximal M-wave measurement. For F- and M-wave measurements, we used an electric stimulator (SEN-8203; Nihon Kohden Corporation, Tokyo, Japan) to deliver square pulse stimuli through a bipolar stimulation probe fixed over the median nerve in the neighborhood of the wrist. The stimulus strength used for F- and M-wave recording was 120% of the stimulus strength required to produce maximum M-wave (*M*_max_), with a pulse duration of 0.2 ms. F-wave amplitude and persistence and M-wave amplitude were measured in 50 trials before and after each task and analyzed.

### Force Recording

For the assessment of muscle fatigue, we measured MVC muscle force before and after each task. We measured thumb opposition movements using a tension gauge (Force Link 9311B; Kistler Japan Co., Ltd., Tokyo, Japan). Maximum muscle force was measured for 5 s and the stable mean of 3 s of this period was calculated.

## Experimental Procedures

**Figure 1:**
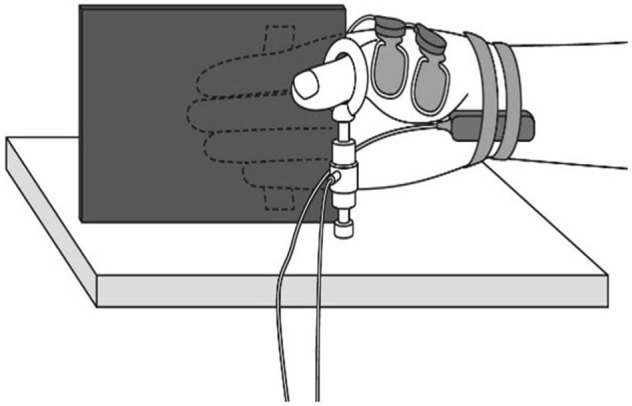
**Muscle force measurement**. During the experiment, participants were comfortably seated with their right shoulder in slight abduction, elbow in 90° flexion, and forearm in a neutral position. A tension gauge was fixed between the metacarpophalangeal and interphalangeal joints of the thumb. The digitus secundus to digitus quintus were fixed to a board mounted on the armchair.

Experiments were performed with three interventions for the same subject. Each sequence was randomized on the other day to avoid influence of the intervention. Participant was seated in a comfortable position and their right arm was fixed to the armchair to allow the measurement of muscle force during thumb opposition movements (Figure 1). The participants performed the following three intervention:

### Intervention 1: Voluntary Contraction (VC) Task

During the VC task, in which muscle fatigue was caused, isometric contraction to achieve thumb opposition was performed for 10 min at an intensity of 10% of MVC. At that time, muscle force was monitored by a personal computer and the participant was instructed to maintain contraction strength using visual feedback.

### Intervention 2: Tetanic ES Task (ES1)

During the ES1 task, in which muscle fatigue was caused, ES was delivered to the median nerve in the wrist (frequency: 20 Hz; duration: 0.2 ms) for 10 min at an intensity of 10% of MVC of thumb opposition.

### Intervention 3: 90% Motor Threshold (MT) ES Task (ES2)

ES2 Task was performed for 8 out of 10 participants to examine whether ES of the degree that muscle fatigue is not caused modulate the excitability of the motor cortex.

During the ES2 task, in which muscle fatigue was not induced, ES was delivered for 10 min at an intensity of 90% of the MT.

**Figure 2:**
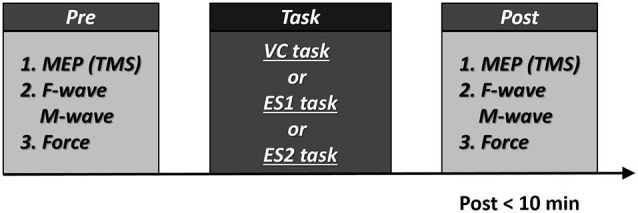
**Experimental procedures 10 participants participated in the experiment.** All participants underwent two tasks [the voluntary contraction (VC) and tetanic electrical stimulation (ES) tasks]. In addition, eight participants performed the 90% motor threshold (MT) ES task. The three tasks were performed in repeated measures design using a randomized order, with a break of at least 1 week between each task. Post-task assessment was measured within 10 min. Numbers represent the order of measurement.

Before each task, we recorded MEP, F-wave and M-wave amplitudes and maximum muscle force. Within 10 min of the end of each task, we recorded MEP, F-wave and M-wave amplitudes and maximum muscle force in the same sequence to allow the comparison of pre- and post-task values (Figure 2). During three tasks, all subjects did not feel pain.

## Statistical Analysis

Statistical analysis were performed using PASW statistics software version 18 (SPSS; IBM, Armonk, NY, USA). Two-way repeated measures analysis of variances [time (pre-intervention, post) × movement type (VC task, ES1 task, and ES2 task)] were used to compare the changes in MEP amplitude, F-wave amplitude and persistence, M-wave amplitude and maximum muscle force. *Post hoc* analysis was performed by Bonferroni’s method. Differences were considered significant at *p* < 0.05 for all analyses.

## Results

### Maximum Muscle Force

**Figure 3:**
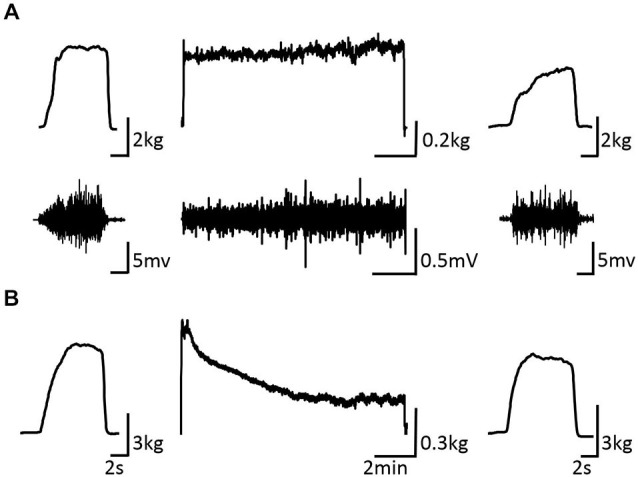
**Pre-, during-, and post-task differences in electromyographic activity. (A)** VC task: the upper stand shows the force waveform, whereas the lower stand shows the electromyographic waveform. **(B)** Tetanic ES task shows the force waveform. It shows the electromyographic activity during the intervention and maximum muscle force pre- and post-intervention of typical subjects.

**Figure 4:**
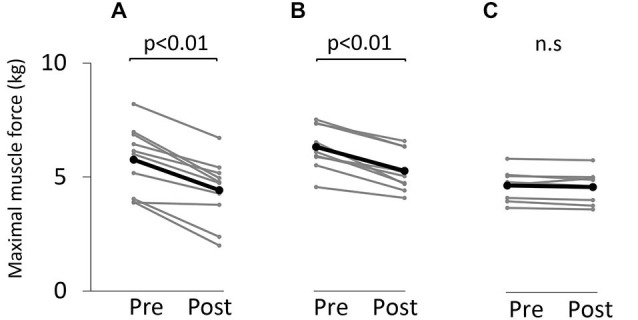
**Maximum muscle force before and after the tasks. (A)** VC task. **(B)** Tetanic ES task. **(C)** The 90% MT ES task. Gray bars indicate the raw waveform of each participant. Black bars indicate the average.

Figure 3 shows the change in maximum muscle force and EMG after the VC and ES1 tasks. Two-way repeated measures analysis of variance revealed significant main effects of time (*F*_(1,25)_ = 91.787, *p* < 0.01), but movement type was not significant (*F*_(2,25)_ = 0.494, *P* > 0.05). The time × movement type interaction was significant (*F*_(2,25)_ = 19.003, *p* < 0.01). *Post hoc* analyses showed that the post-task maximum muscle force for the VC and ES1 tasks was significantly lower than the pre-task value (*p* < 0.01; Figures 4A,B). After the ES2 task, maximum muscle force remained unchanged (Figure 4C; Table 1).

**Table 1:** **Maximum muscle force, motor-evoked potential, F-wave amplitude, F-wave persistence and M-wave amplitude before and after the tasks**.

			VC task (*n* = 10)		ES1 task (*n* = 10)		ES2 task (*n* = 8)	
			Pre	Post	Pre	Post	Pre	Post
**Maximum muscle force**	Amplitude	(kg)	5.76 ± 1.48	4.42 ± 1.41*	5.98 ± 0.88	4.99 ± 0.82*	5.08 ± 0.77	5.00 ± 0.80
**MEP**	Amplitude	(mV)	0.96 ± 0.04	0.67 ± 0.05*	0.94 ± 0.02	0.74 ± 0.03*	0.98 ± 0.05	1.19 ± 0.07
**F-wave**	Amplitude	(mV)	0.37 ± 0.13	0.31 ± 0.08	0.43 ± 0.29	0.36 ± 0.19	0.31 ± 0.10	0.34 ± 0.16
	Persistence	(%)	33.6 ± 13.9	32.0 ± 16.9	25.4 ± 14.1	29.8 ± 13.2	21.0 ± 12.7	18.0 ± 9.7
**M-wave**	Amplitude	(mV)	22.4 ± 6.4	21.7 ± 6.2	20.0 ± 3.9	20.4 ± 5.0	19.8 ± 0.19	0.36 ± 0.19

MEP, Motor-evoked potentials; mean ± standard error. Maximum muscle force, F-wave amplitude, F-wave persistence and M-wave amplitude; mean ± standard deviation. *p < 0.01.

### Motor-Evoked Potential (MEP) Amplitude

**Figure 5:**
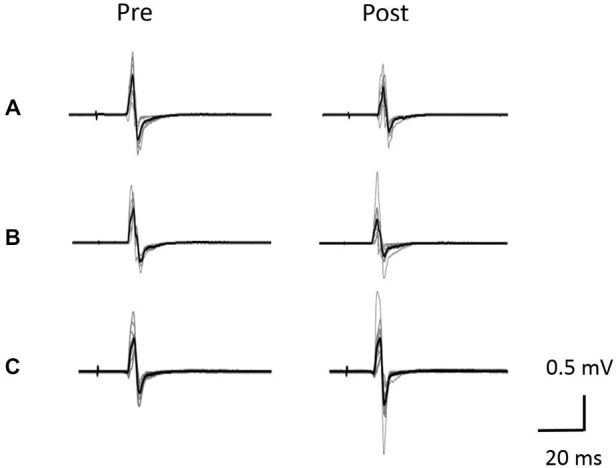
**Typical motor-evoked potential (MEP) waveforms from a representative individual. (A)** VC task. **(B)** Tetanic ES task. **(C)** The 90% MT ES task.Representative superimposed MEP waveforms elicited at the three interventions. MEPs of the right abductor pollicis brevis (APB) were evoked by transcranial magnetic stimulation (TMS) at pre and post-intervention for three interventions. Gray bars indicate the raw waveform. Black bars indicate the mean MEP waveforms.

**Figure 6:**
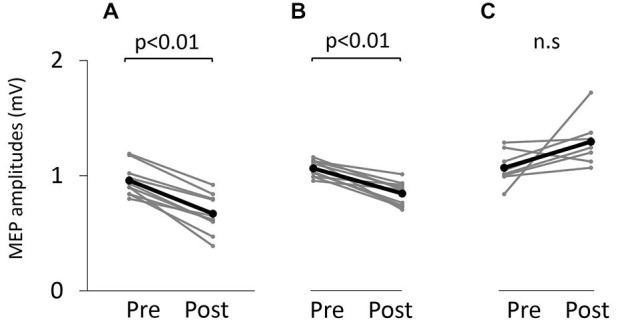
**Motor-evoked potential (MEP) amplitude before and after the tasks. (A)** VC task. **(B)** Tetanic ES task. **(C)** The 90% MT ES task.

Figure 5 shows typical motor-evoked potential (MEP) waveforms from a representative participant. Two-way repeated measures analysis of variance revealed significant main effects of both time (*F*_(1,25)_ = 8.247, *p* < 0.01) and movement type (*F*_(2,25)_ = 17.546, *P* < 0.01). Further, the time × movement type interaction was significant (*F*_(2,25)_ = 21.262, *p* < 0.01). *Post hoc* analyses showed that after the VC and ES1 tasks, MEP amplitude was significantly lower than the pre-task value (*p* < 0.01; Figures 6A,B). After the ES2 task, MEP amplitude remained unchanged (Figure 6C; Table 1).

### M-wave Amplitude

**Figure 7:**
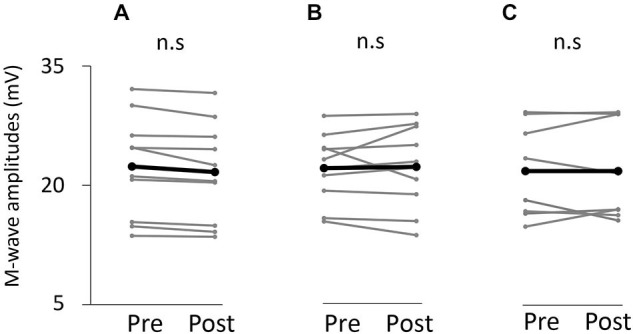
**M-wave amplitude before and after the tasks.** **(A)** VC task. **(B)** Tetanic ES task. **(C)** The 90% MT ES task.

Figure 7 shows information about the M-wave value obtained from each intervention. Two-way repeated measures analysis of variance revealed non-significant main effects of both time (*F*_(1,25)_ = 0.396, *p* > 0.05) and movement type (*F*_(2,25)_ = 0.445, *P* > 0.05). Further, the time × movement type interaction was not significant (amplitude; *F*_(2,25)_ = 0.940, *p* > 0.05).

### F-wave Amplitude and Persistence

Table 1 shows information about the value of the F-wave obtained from each intervention. Two-way repeated measures analysis of variance revealed non-significant main effects of both time (amplitude; *F*_(1,25)_ = 1.761, *p* > 0.05, persistence; *F*_(1,25)_ = 0.001, *p* > 0.05) and movement type (amplitude; *F*_(1,25)_ = 0.489, *P* > 0.05, persistence; *F*_(2,25)_ = 2.360, *P* > 0.05). Further, the time × movement type interaction was not significant (amplitude; *F*_(2,25)_ = 1.614, *P* > 0.05, persistence; *F*_(2,25)_ = 1.749, *P* > 0.05).

## Discussion

Our results indicate that the amplitude of TMS-induced MEPs decreases after tetanic muscle contraction for 10 min, induced by peripheral nerve stimulation, without corresponding changes in supramaximal M- and F-wave amplitude. MEP amplitude also decreases after voluntary fatiguing exercise because of PED. In addition, this MEP depression cannot be induced by ES for 10 min at 90% of the MT without muscle contraction.

The amplitude of MEP induced by TMS in the APB muscle was significantly lower after tetanic muscle contraction elicited by ES of the median nerve for 10 min. Because MVC force decreased after tetanic ES compared with that before tetanic ES, we can assume that the APB muscle was fatigued. Several researchers (Ridding et al., 2000; Kaelin-Lang et al., 2002; Khaslavskaia and Sinkjaer, 2005; Golaszewski et al., 2010, 2012) have shown that MEP amplitude increases after ES. For example, MEP amplitude increases after ES at 50 Hz at the intensity of the sensory threshold, after ES at 2 Hz at the intensity of the MT for 30 min (Golaszewski et al., 2012) and after intermittent ES at 10 Hz for 2 h (Kaelin-Lang et al., 2002). Conversely, some studies (Murakami et al., 2007; Chipchase et al., 2011a) have indicated that MEP amplitude decreases after ES. ES above MT is considered to increase corticomotor excitability, whereas ES below MT produces conflicting results (Chipchase et al., 2011b). The effects of peripheral nerve ES on corticospinal excitability have been thoroughly studied by many research groups, but few studies examine corticospinal excitability after muscle fatigue induced by ES; McKay et al., 1995) demonstrated that MEP amplitude remains unchanged after muscle fatigue induced by intermittent tetanic ES, a result that contradicts our findings. However, these authors used intermittent stimulation at 40 Hz (650 ms on, 350 ms off) for 70 s, whereas we used continuous stimulation at 20 Hz for 10 min. Because PED after voluntary fatiguing exercise is linked to exercise duration (Gandevia et al., 1999), we believe that differences in stimulation time are responsible for this discrepancy.

It is established that Group III and IV muscle afferents fire during continuous muscle contraction in response to both mechanical and metabolic changes in the muscle; these muscle afferents also alter corticospinal excitability (Gandevia et al., 1996; Taylor et al., 1996, 2000b; Ridding and Rothwell, 1997; Ziemann et al., 1998, 2001). Therefore, we can assume that muscle fatigue induced by prolonged tetanic ES in this study increased the activity of Group III and IV muscle afferents and decreased corticospinal excitability. Another of our findings, that MEP was unchanged after continuous ES without muscle contraction, supports the notion that muscle contraction induced by tetanic ES causes MEP depression.

PED occurred after 10% MVC for 10 min without corresponding changes in F- and M-wave amplitude. These results are consistent with previous reports on PED (Brasil-Neto et al., 1993; McKay et al., 1995; Zanette et al., 1995; Samii et al., 1996; Gandevia et al., 1999). Brasil-Neto et al., 1993) described PED after fatiguing voluntary exercise; they found that PED induced by TMS occurs without corresponding changes in M-wave, F-wave or MEP amplitudes induced by transcranial ES. Therefore, it is likely that PED is evidence of the fatigue of motor pathways and reflects changes at the cortical level (Brasil-Neto et al., 1993; McKay et al., 1995; Zanette et al., 1995; Samii et al., 1996; Sacco et al., 1997; Gandevia et al., 1999; Lentz and Nielsen, 2002). Possible explanations for this phenomenon include alteration of the membrane properties of pyramidal cells, a change in synaptic efficacy due to the alteration of neurotransmitter levels (Zanette et al., 1995; Taylor and Gandevia, 2001), a decrease in the excitability of intracortical networks or an increase in the excitability of inhibitory networks (Taylor and Gandevia, 2001; Bonato et al., 2002; Lentz and Nielsen, 2002; Teo et al., 2012). However, the cause of central fatigue remains unclear. It is possible that muscle afferent input to the motor cortex, intracortical input to the motor cortex from a higher level motor-related cortex [e.g., the supplementary motor area (SMA)] or both are responsible for central fatigue. When muscle contractions are maintained at submaximal strength, the increase in effort enhances activity in M1. This increase in effort is considered to reflect an increase in the intensity of corticofugal commands necessary for the maintenance of the intended target force (Liu et al., 2003; van Duinen et al., 2007). van Duinen et al. (2007) showed that activity in SMA increases during fatiguing exercise but that SMA activity during MVC is reduced after fatiguing exercise compared with that before fatigue. Therefore, these authors postulated that SMA functions in the fatigue-related reduction of signals destined for M1 (van Duinen et al., 2007). Nevertheless, after muscle fatigue induced by tetanic ES without voluntary effort, MEP depression was clearly evident in our study. These results suggest that PED is caused by muscle afferents in the muscle becoming fatigued and that voluntary control is not necessarily required for PED.

M-wave amplitude remained unchanged after muscle contraction induced by the VC- and ES1 task, despite existing muscle fatigue. Some studies (Sacco et al., 2000; Lentz and Nielsen, 2002) have reported that M-wave amplitude decreases after muscle fatigue induced by VC. However, several researchers (Brasil-Neto et al., 1993; McKay et al., 1995; Zanette et al., 1995; Löscher et al., 1996) have noted that M-wave amplitude remains unchanged after fatiguing exercise, and our result indicating M-wave amplitude remained unchanged after VS task are in agreement with these previous studies. While peripheral fatigue develops gradually and the recruitment of additional motor units and the firing rate of active motor units increase to sustain the target force during a sustained submaximal isometric contraction, recruitment of additional motor units and an increase in the firing rate do not occur when muscle fatigue is caused by tetanic ES. The number of fatigued muscle fibers is low compared with that during voluntary fatiguing exercise; therefore, it is plausible that M-wave amplitude remains unchanged after fatigue induced by tetanic ES. Accordingly, it is likely that PED occurs at the cortical level, as reported previously (Brasil-Neto et al., 1993; McKay et al., 1995; Zanette et al., 1995; Samii et al., 1996; Gandevia et al., 1999).

α-motoneuron excitability represented by F-wave amplitude and persistence also remained unchanged after fatigue induced by both voluntary and tetanic ES. These results are consistent with previous studies (Brasil-Neto et al., 1993; Zanette et al., 1995; Löscher et al., 1996). However, some studies (McKay et al., 1995) have shown that α-motoneuron excitability decreases after fatiguing exercise. This reduction in α-motoneuron excitability reverts to unfatigued levels within 1 min of the end of fatiguing exercise (McKay et al., 1995). We quantified the F- wave as α-motoneuron excitability after the end of the MEP measurement; accordingly, the F-wave measurement was started at least 1 min after the end of the fatiguing exercise. Therefore, α-motoneuron excitability may not change after muscle fatigue.

In conclusion, our data show that MEP depression occurs after muscle fatigue induced by both tetanic ES and voluntary muscle contraction and that MEP depression does not occur after ES without muscle contraction. These results lead us to hypothesize that central fatigue at the cortical level after voluntary fatiguing exercise is induced by muscle afferents rather than by a voluntary command from a cortical level higher than M1.

## Conflict of Interest Statement

The authors declare that the research was conducted in the absence of any commercial or financial relationships that could be construed as a potential conflict of interest.
